# Supplementary material for: Exploring telerehabilitation awareness, application, and future outlook in sports rehabilitation among physiotherapy students: a web-based survey
Source: PeerJ. 2025 Aug 26;13:e19829. doi: 10.7717/peerj.19829 (PMC12396206; doi:10.7717/peerj.19829)
Supplement: Supplemental Information 1 [file peerj-13-19829-s001.pdf]

QUESTIONNAIRE

Exploring Telerehabilitation Awareness, Application, and Future Outlook in Sports Rehabilitation among Physiotherapy Students: A Web-Based Survey

Demographic Details

Name

Gender

Age

Course

Year of Study

College Name

Screening Part

1. Are you familiar with the term "telerehabilitation" and its applications, such as video calls, mobile apps, and software tools for rehabilitation purposes?

- Yes
- No

2. If yes, where did you first come across the term "telerehabilitation"?

- Academic courses
- Research articles
- Social media
- Workshops or seminars

3. If not, why are you not familiar with the term "telerehabilitation"?

- Lack of exposure/information
- Limited interest in healthcare topics
- Insufficient educational background
- Others

4. Have you received any formal education on telerehabilitation as part of your curriculum?

- Yes
- No

5. Would you be interested in receiving education or training about telerehabilitation techniques as part of your physiotherapy curriculum?

- Yes
- No

6. If "yes," what specific aspects of telerehabilitation education would you find most valuable?

- Practical application of telerehabilitation techniques
- Understanding telecommunication technologies
- Patient assessment and monitoring through telehealth
- Legal and ethical considerations

7. If "no," what factors contribute to your lack of interest in receiving telerehabilitation education as part of your sports study's curriculum?

- Already knowledgeable on the subject
- Prefer traditional in-person instruction
- Not relevant to my career goals
- Lack of time or resources

AWARENESS PART (Strongly Disagree to Strongly Agree)

8. I can differentiate between real-time and store-and-forward telerehabilitation approaches.

9. I can distinguish between telerehabilitation and telemedicine and understand their unique uses.

10.I can identify the common types of sports injuries that are suitable for telerehabilitation.

11. I am aware of the potential role of telerehabilitation in post-operative rehabilitation.
12. I am aware of any professional certification or specialization programs related to telerehabilitation for physiotherapists.
13. The level of support and resources available for students interested in telerehabilitation in physiotherapy program is adequate.
14. I believe that hands-on physiotherapy techniques are essential for effective sports rehabilitation, making telerehabilitation less suitable.
15. Formal education in telerehabilitation would increase my willingness to use it for sports rehabilitation.
16. I am aware of any ongoing research or innovations in the field of telerehabilitation for sports rehabilitation.

17. I perceive telerehabilitation as an environmentally friendly alternative to traditional in-person rehabilitation methods.
18. I am aware of the ethical considerations and guidelines associated with telerehabilitation in sports rehabilitation.

APPLICATION PART (Strongly Disagree to Strongly Agree)

19. Offering telerehabilitation as a treatment option has the potential to reduce the overall cost of sports rehabilitation for athletes and organizations.
20. The availability of resources, including technology, affects the effectiveness of telerehabilitation in sports rehabilitation.
21. I think that telerehabilitation has the potential to bridge geographical gaps in providing care to athletes, enabling remote areas to access high-quality physiotherapy services.
22. I believe effective communication with athletes and motivation for adherence to their rehabilitation programs is assured in telerehabilitation.
23. I believe that telerehabilitation could be integrated with virtual reality to create more engaging and interactive rehabilitation experiences for athletes.
24. I believe Telerehabilitation achieve the same outcomes as in-person rehabilitation.
25. I believe that athletes benefit from quicker access to telerehabilitation services for their sports injuries.
26. I believe that tele-rehabilitation could contribute to a more sustainable approach to sports rehabilitation by reducing the need for travel and physical resources.
27. I'm open to considering telerehabilitation as a way to improve access to sports rehabilitation expertise for amateur athletes and those in underprivileged communities.
28. I believe that ongoing research and evidence-based practice are essential for the successful implementation of telerehabilitation in sports rehabilitation.
29. Establishing a specialized field for telerehabilitation within the discipline is something that can be considered.
30. I think telerehabilitation can help in tracking and monitoring an athlete's progress during the rehabilitation process?
31. I agree that telerehabilitation can play a significant role in reducing the recovery time for athletes with sports injuries.

Future Outlook Part (Strongly Disagree to Strongly Agree)

32. I am willing to invest time and effort to stay updated on the latest advancements in telerehabilitation techniques.
33. I think that telerehabilitation will become a standard practice in the field of sports rehabilitation.
34. I believe that integrating telerehabilitation into the curriculum of physiotherapy programs is essential for future professionals.
35. I am confident that telerehabilitation will continue to gain acceptance and recognition within the healthcare industry for sports rehabilitation.
36. I believe that telerehabilitation will continue to evolve and address the challenges and limitations associated with sports rehabilitation.
37. I believe that telerehabilitation could be used to create personalized, AI-guided warm-up and cool-down routines for athletes.
38. I believe that telerehabilitation could employ elements of neurofeedback to enhance an athlete's mental resilience during recovery.
39. Your level of awareness regarding regulatory or legal aspects of telerehabilitation can impact your readiness to integrate it into your sports rehabilitation practice.
40. I believe that telerehabilitation will encourage athletes to take a more active role in their rehabilitation and recovery.
41. I agree that technical issues, such as poor internet connectivity, can hinder the effectiveness of telerehabilitation.
42. I agree that difficulty in accurately assessing the progress of athletes during telerehabilitation is a challenge.
