# Supplementary material for: Exploring telerehabilitation awareness, application, and future outlook in sports rehabilitation among physiotherapy students: a web-based survey
Source: PeerJ. 2025 Aug 26;13:e19829. doi: 10.7717/peerj.19829 (PMC12396206; doi:10.7717/peerj.19829)
Supplement: Supplemental Information 3 [file peerj-13-19829-s003.docx]

**Exploring Telerehabilitation Awareness, Application, and Future Outlook in Sports Rehabilitation among Physiotherapy Students: A Web-Based Survey**

**Self-Explanatory Note for Raw Data**

**Survey Overview:**

This study explores the awareness, practical application knowledge, and future potential of telerehabilitation in sports rehabilitation among physiotherapy students. Using a web-based survey, the research captures students' familiarity with telerehabilitation tools, their perceptions of its role in remote care, and the challenges they foresee in its implementation.

The complete questionnaire is available in Supplementary Material 1.

The colour flow of the questionnaire can be identified in the raw data (Supplementary File 2).

**Key Variables**

**Demographics**

- **ID**: Unique identifier for each participant.
- **Gender**: Participant's gender (Male/Female).
- **Age**: Age group of the participant.
- **Course**: Academic program (UG – Undergraduate, PG – Postgraduate, Ph.D.,)
- **Year**: Year of study in the academic program.
- **Region**: Geographical location within Country Participants (e.g., South India, North India).
- **Inside or Outside India**: Participant's region (INSIDE INDIA / OUTSIDE INDIA).

**Questionnaire Structure and Flow:**

**1. Screening Questions (Q1 to Q7) - Yellow Section:**

- This section explores participants' basic knowledge about telerehabilitation.
- The primary purpose is to determine whether participants have sufficient foundational knowledge to proceed with the survey.

**Key Screening Flow:**

- **Q1:** Assesses participants' basic knowledge about telerehabilitation.
  - If the participant answers "Yes": They proceed to Q2 to specify where they first came across the term "telerehabilitation." After answering Q2, they continue to the q4, q5 and main survey sections (Awareness, Application, and Future Outlook).
  - If the participant answers "No": They are directed to the final question of the screening section (Q3) to specify their reason for lack of awareness about telerehabilitation. The participant's responses are then submitted without further continuation of the survey.
- **Q5:** Assesses participants' interest in receiving education or training about telerehabilitation techniques as part of their physiotherapy curriculum.
  - If "Yes": Participants are directed to **Q6** to elaborate on the specific aspects of telerehabilitation education they find most valuable.
  - If "No": Participants are directed to **Q7** to identify the factors contributing to their lack of interest in telerehabilitation education.
- After completing the screening section, participants who meet the criteria (answered "Yes" to Q1) move forward to the main survey.

**2. Awareness Questions (Q8 to Q18) - Green Section:**

- This section examines participants' current understanding and knowledge of telerehabilitation.
- Questions focus on identifying awareness levels and familiarity with key concepts, benefits, and challenges associated with telerehabilitation.

**3. Application Questions (Q19 to Q31) - Blue Section:**

- This section evaluates participants' ability to apply telerehabilitation knowledge in sports rehabilitation scenarios.
- Questions explore how participants perceive telerehabilitation’s practical implementation, potential use cases, and real-world applications in physiotherapy practice.

**4. Future Outlook Questions (Q32 to Q42) - Grey Section:**

- This section investigates participants' perspectives on the future of telerehabilitation in sports rehabilitation.
- Questions focus on expectations, potential advancements, and the role of telerehabilitation in shaping the future of physiotherapy practice.

**EXPLANATION OF QUESTIONNAIRE FOR REPRODUCIBILITY**

**Screening Section (Q1 to Q7):**

- Q1 is a critical filter question. It determines whether the participant is aware of telerehabilitation. If the participant answers "No," they skip directly to Q3, where they provide their reason for lack of awareness. After answering Q3, their response is submitted, and they do not proceed further in the survey.
- If participants answer "Yes" to Q1, they continue with the rest of the survey.
- Q5 identifies interest in telerehabilitation education:
  - If "Yes," participants proceed to Q6 to specify the aspects of education they value.
  - If "No," participants proceed to Q7 to identify reasons for their lack of interest.

**Main Survey (Q8 to Q42):**

Participants who pass the screening section proceed to the main survey, which is divided into three parts:

1. **Awareness Questions (Q8 to Q18):**
   - Explore participants' current knowledge and familiarity with telerehabilitation concepts.
   - Example: Understanding the benefits of telerehabilitation in remote sports injury management.
2. **Application Questions (Q19 to Q31):**
   - Assess participants' ability to apply telerehabilitation knowledge in practical settings.
   - Example: Identifying how telerehabilitation can enhance patient outcomes in sports rehabilitation.
3. **Future Outlook Questions (Q32 to Q42):**
   - Gauge participants' perceptions of telerehabilitation’s potential in advancing physiotherapy.
   - Example: Exploring anticipated advancements in telerehabilitation technology and its integration into routine practice.
