# Supplementary material for: Exploring telerehabilitation awareness, application, and future outlook in sports rehabilitation among physiotherapy students: a web-based survey
Source: PeerJ. 2025 Aug 26;13:e19829. doi: 10.7717/peerj.19829 (PMC12396206; doi:10.7717/peerj.19829)
Supplement: Supplemental Information 5 [file peerj-13-19829-s005.docx]

| **TELEREHABILITATION AWARENESS** | **Strongly Agree**  **n (%)** | **Agree**  **n (%)** | **Neutral**  **n (%)** | **Disagree**  **n (%)** | **Strongly Disagree**  **n (%)** |
| --- | --- | --- | --- | --- | --- |
| I can differentiate between real-time and store-and-forward telerehabilitation approaches. | 93 (27.2%) | 73 (21.3%) | 83 (24.3%) | 21 (6.1%) | 72 (21.1%) |
| I can distinguish between telerehabilitation and telemedicine and understand their unique uses. | 121 (35.4%) | 88 (25.7%) | 58 (17.0%) | 16 (4.7%) | 59 (17.3%) |
| I can identify the common types of sports injuries that are suitable for telerehabilitation. | 113 (33.0%) | 78  (22.8%) | 61 (11.6%) | 32 (17.8%) | 58 (17.0%) |
| I am aware of the potential role of telerehabilitation in post-operative rehabilitation. | 116 (33.9%) | 88 (25.7%) | 57 (16.7%) | 26 (7.6%) | 55 (16.1%) |
| I am aware of any professional certification or specialization programs related to telerehabilitation for physiotherapists. | 77 (22.5%) | 70 (20.5%) | 58 (17.0%) | 76 (22.2%) | 61 (17.8%) |
| The level of support and resources available for students interested in telerehabilitation in physiotherapy program is adequate. | 72 (21.1%) | 62 (18.1%) | 67 (19.6%) | 67 (19.6%) | 74(21.6%) |
| I believe that hands-on physiotherapy techniques are essential for effective sports rehabilitation, making telerehabilitation less suitable. | 133 (38.9%) | 92 (26.9%) | 55 (16.1%) | 20 (5.8%) | 42 (12.3%) |
| Formal education in telerehabilitation would increase my willingness to use it for sports rehabilitation. | 86 (25.1%) | 141 (41.2%) | 41 (12.0%) | 12 (3.5%) | 62 (18.8%) |
| I am aware of any ongoing research or innovations in the field of telerehabilitation for sports rehabilitation. | 93 (27.2%) | 64 (18.7%) | 64 (18.7%) | 60 (17.5%) | 61 (17.8%) |
| I perceive telerehabilitation as an environmentally friendly alternative to traditional in-person rehabilitation methods. | 109 (31.9%) | 78 (22.8%) | 65 (19.0%) | 23 (6.7%) | 67 (19.6%) |
| I am aware of the ethical considerations and guidelines associated with telerehabilitation in sports rehabilitation. | 92 (26.9%) | 58 (17.0%) | 68 (19.9%) | 62 (18.1%) | 62 (18.1%) |
