# Supplementary material for: Exploring telerehabilitation awareness, application, and future outlook in sports rehabilitation among physiotherapy students: a web-based survey
Source: PeerJ. 2025 Aug 26;13:e19829. doi: 10.7717/peerj.19829 (PMC12396206; doi:10.7717/peerj.19829)
Supplement: Supplemental Information 6 [file peerj-13-19829-s006.docx]

| **Variable** | **AWARENESS** | | | | | **X^2^** | **Strength of Association**  **(φ & Vc)** | ***P* Value** |
| --- | --- | --- | --- | --- | --- | --- | --- | --- |
|  | **Strongly Agree** | **Agree** | **Neutral** | **Disagree** | **Strongly Disagree** |  |  |  |
| **Age** |  | | | | | | | |
| 18-20 | 29 | 46 | 17 | 02 | 01 | 27.541 | 0.284 | 0.001 |
| 21-25 | 48 | 55 | 71 | 20 | 09 |  |  |  |
| >25 | 15 | 09 | 15 | 03 | 02 |  |  |  |
| **Gender** | | | | | | 8.180 | 0.155 | 0.085 |
| Female | 51 | 51 | 65 | 15 | 09 |  |  |  |
| Male | 41 | 59 | 38 | 10 | 03 |  |  |  |
| **Academic Level** | | | | | | | | |
| UG | 66 | 77 | 61 | 14 | 11 | 13.798 | 0.142 | 0.087 |
| PG | 19 | 28 | 33 | 11 | 1 |  |  |  |
| Ph.D., | 07 | 05 | 09 | 0 | 0 |  |  |  |
| **Region** | | | | | | | | |
| Domestic Realm (India) | 78 | 96 | 78 | 16 | 08 | 11.665 | 0.185 | 0.020 |
| Global Realm | 14 | 14 | 25 | 09 | 04 |  |  |  |
