# Supplementary material for: Exploring telerehabilitation awareness, application, and future outlook in sports rehabilitation among physiotherapy students: a web-based survey
Source: PeerJ. 2025 Aug 26;13:e19829. doi: 10.7717/peerj.19829 (PMC12396206; doi:10.7717/peerj.19829)
Supplement: Supplemental Information 7 — Impact of Demographic Predictors on Telerehabilitation Awareness and Application through Ordinal Logistic Regression Analysis [file peerj-13-19829-s007.docx]

| **Demographics** | **AWARENESS** | | | | **APPLICATION** | | | |
| --- | --- | --- | --- | --- | --- | --- | --- | --- |
|  | **Regression Coefficient** | ***P* Value** | **OR** | **95% CI for OR** | **Regression Coefficient** | ***P* Value** | **OR** | **95% CI for OR** |
| **Gender** | | | | | | | | |
| Female | -0.29 | 0.152 | 0.748 | 0.503,1.113 | -0.228 | 0.261 | 0.796 | 0.535, 1.185 |
| Male | 0.000 |  | 1.000 |  | 0.000 |  | 1.000 |  |
| **AGE** | | | | | | | | |
| 18-20 | 0.472 | 0.260 | 1.602 | 0.705, 3.642 | -1.509 | 0.000 | 0.221 | 0.95, 0.513 |
| 21-25 | -0.210 | 0.570 | 0.811 | 0.393, 1.673 | -0.339 | 0.367 | 0.712 | 0.341, 1.488 |
| >25 | 0.000 |  | 1.000 |  | 0.000 |  | 1.000 |  |
| **Academic Level** | | | | | | | | |
| UG | -0.173 | 0.739 | 0.841 | 0.303, 2.332 | -0.149 | 0.779 | .862 | 0.306, 2.429 |
| PG | -0.403 | 0.439 | 0.668 | 0.240, 1.857 | -0.522 | 0.324 | .593 | 0.210, 1.676 |
| Ph.D., | 0.000 |  | 1.000 |  | 0.000 |  | 1.000 |  |
| **REGION** | | | | | | | | |
| Domestic Realm (India) | 0.669 | 0.013 | 1.953 | 1.153, 3.308 | -0.305 | 0.259 | 0.737 | 0.435, 1.251 |
| Global Realm | 0.000 |  | 1.000 |  | 0.000 |  | 1.000 |  |
