# Supplementary material for: Exploring telerehabilitation awareness, application, and future outlook in sports rehabilitation among physiotherapy students: a web-based survey
Source: PeerJ. 2025 Aug 26;13:e19829. doi: 10.7717/peerj.19829 (PMC12396206; doi:10.7717/peerj.19829)
Supplement: Supplemental Information 8 [file peerj-13-19829-s008.docx]

| **APPLICATION OF TELEREHABILITATION** | **Strongly Agree**  **n (%)** | **Agree**  **n (%)** | **Neutral**  **n (%)** | **Disagree**  **n (%)** | **Strongly Disagree**  **n (%)** |
| --- | --- | --- | --- | --- | --- |
| Offering telerehabilitation as a treatment option has the potential to reduce the overall cost of sports rehabilitation for athletes and organizations. | 108 (31.6%) | 82 (24.0%) | 60 (17.5%) | 12 (3.5%) | 80 (23.4%) |
| The availability of resources, including technology, affects the effectiveness of telerehabilitation in sports rehabilitation. | 113 (33.0%) | 87 (25.4%) | 56 (16.4%) | 14 (4.1%) | 72 (21.1%) |
| I think that telerehabilitation has the potential to bridge geographical gaps in providing care to athletes, enabling remote areas to access high-quality physiotherapy services. | 129 (37.7%) | 131 (38.3%) | 36 (10.5%) | 10 (2.9%) | 36 (10.5%) |
| I believe effective communication with athletes and motivation for adherence to their rehabilitation programs is assured in telerehabilitation. | 152 (44.4%) | 118 (34.5%) | 12 (3.5%) | 7 (2.0%) | 53 (15.5%) |
| I believe that telerehabilitation could be integrated with virtual reality to create more engaging and interactive rehabilitation experiences for athletes. | 124 (36.3%) | 101 (29.5%) | 47 (13.7%) | 9 (2.6%) | 61 (17.8%) |
| I believe Telerehabilitation achieve the same outcomes as in-person rehabilitation. | 74 (21.6%) | 50 (14.6%) | 71 (20.8%) | 64 (18.7%) | 83 (24.3%) |
| I believe that athletes benefit from quicker access to telerehabilitation services for their sports injuries. | 110 (32.2%) | 78 (22.8%) | 58 (17.0%) | 22 (6.4%) | 74 (21.6%) |
| I believe that tele-rehabilitation could contribute to a more sustainable approach to sports rehabilitation by reducing the need for travel and physical resources. | 120 (35.1%) | 131 (38.3%) | 6 (1.8%) | 17 (5.0%) | 68 (19.9%) |
| I'm open to considering telerehabilitation as a way to improve access to sports rehabilitation expertise for amateur athletes and those in underprivileged communities. | 60 (17.5%) | 127 (37.1%) | 52 (15.2%) | 14 (4.1%) | 89 (26.0%) |
| I believe that ongoing research and evidence-based practice are essential for the successful implementation of telerehabilitation in sports rehabilitation. | 66 (19.3%) | 153 (44.7%) | 43 (12.6%) | 10 (2.9%) | 70 (20.5%) |
| Establishing a specialized field for telerehabilitation within the discipline is something that can be considered. | 102 (29.8%) | 98 (28.7%) | 49 (14.3%) | 11 (3.2%) | 82 (24.0%) |
| I think telerehabilitation can help in tracking and monitoring an athlete's progress during the rehabilitation process? | 136 (39.8%) | 102 (29.8%) | 36 (10.5%) | 12 (3.5%) | 56 (16.4%) |
| I agree that telerehabilitation can play a significant role in reducing the recovery time for athletes with sports injuries. | 111 (32.5%) | 80 (23.4%) | 53 (15.5%) | 22 (6.4%) | 76 (22.2%) |
