# Supplementary material for: Exploring telerehabilitation awareness, application, and future outlook in sports rehabilitation among physiotherapy students: a web-based survey
Source: PeerJ. 2025 Aug 26;13:e19829. doi: 10.7717/peerj.19829 (PMC12396206; doi:10.7717/peerj.19829)
Supplement: Supplemental Information 9 [file peerj-13-19829-s009.docx]

| **Variable** | **APPLICATION KNOWLEDGE** | | | | | **χ²** | **Strength of Association**  **(φ & Vc)** | ***P* Value** |
| --- | --- | --- | --- | --- | --- | --- | --- | --- |
|  | **Strongly Agree** | **Agree** | **Neutral** | **Disagree** | **Strongly Disagree** |  |  |  |
| **Age** |  | | | | | | | |
| 18-20 | 13 | 23 | 27 | 19 | 13 | 35.215 | 0.227 | 0.000 |
| 21-25 | 40 | 92 | 47 | 14 | 10 |  |  |  |
| >25 | 13 | 21 | 07 | 02 | 01 |  |  |  |
| **Gender** | | | | | | 12.272 | 0.189 | 0.015 |
| Female | 40 | 63 | 55 | 17 | 16 |  |  |  |
| Male | 26 | 73 | 26 | 18 | 08 |  |  |  |
| **Academic Level** | | | | | | | | |
| UG | 42 | 93 | 47 | 29 | 18 | 13.603 | 0.141 | 0.093 |
| PG | 19 | 31 | 30 | 06 | 06 |  |  |  |
| Ph.D., | 05 | 12 | 04 | 0 | 0 |  |  |  |
| **Region** | | | | | | | | |
| Domestic Realm (India) | 47 | 109 | 70 | 31 | 19 | 6.972 | 0.143 | 0.137 |
| Global Realm | 19 | 27 | 11 | 04 | 05 |  |  |  |
