# Supplementary material for: Exploring telerehabilitation awareness, application, and future outlook in sports rehabilitation among physiotherapy students: a web-based survey
Source: PeerJ. 2025 Aug 26;13:e19829. doi: 10.7717/peerj.19829 (PMC12396206; doi:10.7717/peerj.19829)
Supplement: Supplemental Information 10 [file peerj-13-19829-s010.docx]

| **FUTURE OUTLOOK OF TELEREHABILITATION** | **Strongly Agree**  **n (%)** | **Agree**  **n (%)** | **Neutral**  **n (%)** | **Disagree**  **n (%)** | **Strongly Disagree**  **n (%)** |
| --- | --- | --- | --- | --- | --- |
| I am willing to invest time and effort to stay updated on the latest advancements in telerehabilitation techniques. | 104 (30.4%) | 89 (26.0%) | 99 (28.9%) | 9 (2.6%) | 41 (12.0%) |
| I think that telerehabilitation will become a standard practice in the field of sports rehabilitation. | 85 (24.9%) | 69 (20.2%) | 131 (38.3%) | 24 (7.0%) | 33 (9.6%) |
| I believe that integrating telerehabilitation into the curriculum of physiotherapy programs is essential for future professionals. | 97 (28.4%) | 100 (29.2%) | 93 (27.2%) | 5 (1.5%) | 47 (13.7%) |
| I am confident that telerehabilitation will continue to gain acceptance and recognition within the healthcare industry for sports rehabilitation. | 119 (34.8%) | 88 (25.7%) | 49 (14.3%) | 10 (2.9%) | 76 (22.2%) |
| I believe that telerehabilitation will continue to evolve and address the challenges and limitations associated with sports rehabilitation. | 122 (35.7%) | 86 (25.1%) | 43 (12.6%) | 9 (2.6%) | 82 (24.0%) |
| I believe that telerehabilitation could be used to create personalized, AI-guided warm-up and cool-down routines for athletes. | 100 (29.2%) | 98 (28.7%) | 91 (26.6%) | 10 (2.9%) | 43 (12.6%) |
| I believe that telerehabilitation could employ elements of neurofeedback to enhance an athlete's mental resilience during recovery. | 84 (24.6%) | 73 (21.3%) | 139 (40.6%) | 18 (5.3%) | 28 (8.2%) |
| Your level of awareness regarding regulatory or legal aspects of telerehabilitation can impact your readiness to integrate it into your sports rehabilitation practice. | 76 (22.2%) | 76 (22.2%) | 148 (43.3%) | 1 (0.3%) | 41 (12.0%) |
| I believe that telerehabilitation will encourage athletes to take a more active role in their rehabilitation and recovery. | 94 (27.5%) | 78 (22.8%) | 120 (35.1%) | 14 (4.1%) | 36 (10.5%) |
| I agree that technical issues, such as poor internet connectivity, can hinder the effectiveness of telerehabilitation. | 104 (30.4%) | 105 (30.7%) | 83 (24.3%) | 6 (1.8%) | 44 (12.9%) |
| I agree that difficulty in accurately assessing the progress of athletes during telerehabilitation is a challenge. | 100 (29.2%) | 104 (30.4%) | 92 (26.9%) | 2 (0.6%) | 44 (12.9%) |
