# Supplementary material for: Exploring telerehabilitation awareness, application, and future outlook in sports rehabilitation among physiotherapy students: a web-based survey
Source: PeerJ. 2025 Aug 26;13:e19829. doi: 10.7717/peerj.19829 (PMC12396206; doi:10.7717/peerj.19829)
Supplement: Supplemental Information 11 [file peerj-13-19829-s011.docx]

| **Variable** | **FUTURE OUTLOOK** | | | | | **X^2^** | **Strength of Association** | ***P* Value** |
| --- | --- | --- | --- | --- | --- | --- | --- | --- |
|  | **Strongly Agree** | **Agree** | **Neutral** | **Disagree** | **Strongly Disagree** |  |  |  |
| **Age** |  | | | | | | | |
| 18-20 | 15 | 25 | 31 | 07 | 17 | 31.621 | 0.215 | 0.000 |
| 21-25 | 57 | 83 | 31 | 06 | 26 |  |  |  |
| >25 | 16 | 20 | 07 | 0 | 01 |  |  |  |
| **Gender** | | | | | | 6.831 | 0.141 | 0.145 |
| Female | 50 | 67 | 47 | 67 | 50 |  |  |  |
| Male | 38 | 61 | 22 | 61 | 38 |  |  |  |
| **Academic Level** | | | | | | | | |
| UG | 54 | 91 | 49 | 10 | 25 | 9.972 | 0.121 | 0.267 |
| PG | 28 | 28 | 15 | 03 | 18 |  |  |  |
| Ph.D., | 06 | 09 | 05 | 0 | 01 |  |  |  |
| **Region** | | | | | | | | |
| Domestic Realm (India) | 71 | 106 | 60 | 08 | 31 | 8.131 | 0.154 | 0.087 |
| Global Realm | 17 | 22 | 09 | 05 | 13 |  |  |  |
