# Supplementary material for: Exploring telerehabilitation awareness, application, and future outlook in sports rehabilitation among physiotherapy students: a web-based survey
Source: PeerJ. 2025 Aug 26;13:e19829. doi: 10.7717/peerj.19829 (PMC12396206; doi:10.7717/peerj.19829)
Supplement: Supplemental Information 12 [file peerj-13-19829-s012.docx]

| **Future Outlook** | | **Regression Coefficient** | **Wald** | ***P***  **Value** | **OR** | **95% CI for OR** | |
| --- | --- | --- | --- | --- | --- | --- | --- |
|  |  |  |  |  |  | **Lower Bound** | **Upper Bound** |
| **Low** | Intercept | -1.208 | 0.821 | 0.365 |  |  |  |
|  | **Gender** | | | | | | |
|  | Female | -0.866 | 6.099 | 0.014 | 0.420 | 0.211 | 0.836 |
|  | Male | 0 |  |  |  |  |  |
|  | **Age** | | | | | | |
|  | 18-20 | 3.423 | 7.269 | 0.007 | 30.676 | 2.547 | 369.490 |
|  | 21-25 | 2.697 | 5.133 | 0.023 | 14.840 | 1.439 | 153.047 |
|  | >25 | 0 |  |  |  |  |  |
|  | **Academic Level** | | | | | | |
|  | UG | -1.255 | 0.932 | 0.334 | 0.285 | 0.022 | 3.643 |
|  | PG | 0.109 | 0.008 | 0.931 | 1.116 | 0.095 | 13.137 |
|  | Ph.D., | 0 |  |  |  |  |  |
|  | **Region** | | | | | | |
|  | Domestic Realm (India) | -1.424 | 9.740 | 0.002 | 0.241 | 0.098 | 0.589 |
|  | Global Realm | 0 |  |  |  |  |  |
| **High** | Intercept | -0.141 | 0.054 | 0.816 |  |  |  |
|  | **Gender** | | | | | | |
|  | Female | -0.527 | 3.963 | 0.047 | 0.590 | 0.351 | 0.992 |
|  | Male | 0 |  |  |  |  |  |
|  | **Age** | | | | | | |
|  | 18-20 | -1.810 | 10.681 | 0.001 | 0.164 | 0.055 | 0.484 |
|  | 21-25 | -0.263 | 0.308 | 0.579 | 0.769 | 0.304 | 1.946 |
|  | >25 | 0 |  |  |  |  |  |
|  | **Academic Level** | | | | | | |
|  | UG | 1.056 | 2.716 | 0.099 | 2.875 | 0.819 | 10.095 |
|  | PG | 0.524 | 0.688 | 0.407 | 1.689 | 0.490 | 5.823 |
|  | Ph.D., | 0 |  |  |  |  |  |
|  | **Region** | | | | | | |
|  | Domestic Realm (India) | 0.733 | 3.984 | 0.046 | 2.082 | 1.013 | 4.277 |
|  | Global Realm | 0 |  |  |  |  |  |
